# Predictive value of peripheral regulatory T cells in non-small cell lung cancer patients undergoing radiotherapy

**DOI:** 10.18632/oncotarget.15238

**Published:** 2017-02-09

**Authors:** Chao Liu, Shikai Wu, Xiangying Meng, Guangxian Liu, Dongmei Chen, Yang Cong, Ge Shen, Bing Sun, Wei Wang, Qian Wang, Hongjun Gao, Xiaoqing Liu

**Affiliations:** ^1^ Department of Radiation Oncology, Affiliated Hospital of Academy of Military Medical Sciences, Beijing, China; ^2^ Cancer Therapy Center, Affiliated Hospital of Academy of Military Medical Sciences, Beijing, China; ^3^ Department of Lung Cancer, Affiliated Hospital of Academy of Military Medical Sciences, Beijing, China

**Keywords:** T cells, lymphocyte subsets, stereotactic body radiation therapy, non-small cell lung cancer, immunotherapy

## Abstract

**Background:**

Studies increasingly focus on the impact of radiotherapy on immunity; however, the role of peripheral cellular immunity prior to radiotherapy in cancer patients remains largely unknown. In this study, we investigated the predictive roles of lymphocyte subsets on tumor progression in non-small cell lung cancer (NSCLC) patients undergoing radiotherapy, and their expression in NSCLC patients at first relapse.

**Methods:**

We enrolled 70 NSCLC patients and 14 age- and sex-matched healthy donors and tested the lymphocyte subsets in their peripheral blood by flow cytometry. Among them, 40 newly diagnosed patients received radiotherapy and were enrolled to investigate the predictive value of lymphocyte subsets on tumor progression after radiotherapy by uni- and multivariate analyses; 30 patients at first relapse were included to evaluate the differences of lymphocyte subsets between them and first diagnosed patients and healthy volunteers.

**Results:**

Increased proportions of regulatory T cells, CD8+ T cells, and CD8+CD28- T cells and decreased CD4+ T cells and CD4/CD8 ratios were observed in NSCLC patients at first relapse compared to newly diagnosed patients. In the 40 first diagnosed patients undergoing radiotherapy, uni- and multivariate analyses showed that increased level of regulatory T cells correlated with poor progression-free survival (hazard ratio = 2.55 and 3.76, *P* = 0.022 and 0.010, respectively).

**Conclusions:**

Peripheral regulatory T cells were increased and independently predict tumor progression in NSCLC patients undergoing radiotherapy, suggesting the promising combination of radiotherapy and immunotherapy.

## INTRODUCTION

As the most common malignant tumor worldwide, approximately 80% of lung cancer patients are non-small cell lung cancer (NSCLC) cases [1, 2]. In recent years, radiotherapy lonely and in combination with other therapies are used as prevailing treatments in ~ 60% of newly diagnosed NSCLC patients [3]. Stereotactic body radiotherapy (SBRT) is becoming as the predominant treatment for NSCLC, especially for early stage ones [4, 5]. Besides the effect of direct killing, radiation induces immunologic response and consequently eliminates non-irradiated tumor cells [6, 7]. According to the immune mechanism of radiotherapy, we proposed that the immune status before radiotherapy may impact the tumor response to radiation and accordingly affect long-term survival. Thus, we aimed to evaluate the clinical values of pretreatment immune cells in peripheral blood in NSCLC patients undergoing radiation therapy.

The theory of cancer immunoediting, including immune elimination, equilibrium, and escape, describes the dual role of immunity in tumor suppression and protection. Immune function in cancer patients closely relates to cancer occurrence and progression [8]. Lymphocytes are significant components of human cellular immunity; different lymphocyte subsets have been observed according to different phenotypes and functions [9]. Several studies have investigated the expression of peripheral lymphocyte subsets in NSCLC. For example, decreased proportions of CD4+ cells (T helper cells), CD4/CD8 ratios, and B cells and increased CD8+CD28- T lymphocytes and regulatory T (Treg) cells were observed in patients with lung cancer [10–13].

In addition to the expression of lymphocyte subsets, researchers also tested their predictive and prognostic roles in NSCLC. Many studies proved the prognostic value of tumor-infiltrating lymphocytes of different subtypes, including CD3+, CD4+, CD8+, Treg cells [14–18]. However, the predictive roles of lymphocyte subpopulations in peripheral blood were not well studied. McCoy et al. [19] found that an elevated proportion of peripheral Treg cells was associated with poor survival in patients with thoracic malignancies. A very recent study has demonstrated that the prognostic value of CD4+ Treg subtypes in NSCLC patients received chemotherapy [20]. Nevertheless, in NSCLC patients undergoing radiotherapy, the clinical significance of lymphocyte subsets is poorly investigated.

The present study aimed to evaluate the predictive value of Treg cells and other lymphocyte subsets in NSCLC patients treated with radiotherapy, and their expression in NSCLC patients at first relapse.

## RESULTS

### Baseline characteristics

Table 1 shows the clinicopathologic characteristics of 70 NSCLC patients. The mean age of 70 NSCLC patients was 63 (range 36-90). No differences of baseline characteristics were observed between patients at first diagnosis and relapse, providing an opportunity to compare the expression of lymphocyte subsets between them. Among 40 newly diagnosed patients, 24 (60%) were treated with SBRT with 30-50 Gy/5 fractions; 16 (40%) received conventional fraction radiotherapy with 60-66 Gy/30-33 fractions for their lung masses. Twenty-two patients underwent positron emission tomography-computed tomography before treatment (data not shown); mean standard uptake value (SUV) of lung mass was 11.1 (range, 3.2-30.8). There were 3 patients with epidermal growth factor receptor mutation and 2 patients with anaplastic lymphoma kinase gene fusion and KRAS mutation, respectively.

**Table 1:** Comparisons of baseline characteristics between NSCLC-D and NSCLC-R

NSCLC Patients (*N*=70)			
Characteristic	NSCLC-D (*N*= 40)	NSCLC-R (*N*= 30)	*P*
	*N*(%)	*N*(%)	
**Sex**			
Male	28(70)	22(73.3)	0.760
Female	12(30)	8(26.7)	
**Age(years)**			
<60	14(35)	15(50)	0.207
≥60	26(65)	15(50)	
**Smoking**			
Never	14(35)	11(36.7)	0.688
Previous	3(7.5)	4(13.3)	
Present	23(57.5)	15(50)	
**ECOG PS**			
0	16(40)	10(33.3)	0.669
1	21(52.5)	19(63.3)	
2	3(7.5)	1(3.3)	
**Histology**			
SCC	19(47.5)	15(50)	0.929
ADC	19(47.5)	13(43.3)	
Other	2(5)	2(6.7)	
**Differentiation**			
Well	3(7.5)	2(6.7)	0.925
Moderate	21(52.5)	15(50)	
Poor	9(22.5)	9(30)	
Unknown	7(17.5)	4(13.3)	
**Gene mutation**			
Yes	5(12.5)	5(16.7)	0.906
No	32(80)	23(76.7)	
Unknown	3(7.5)	2(6.7)	
**Tumor stage**			
T1	10(25)	6(20)	0.835
T2	17(42.5)	12(40)	
T3	6(15)	7(23.3)	
T4	7(17.5)	5(16.7)	
**Nodal stage**			
N0	11(27.5)	7(23.3)	0.639
N1	11(27.5)	7(23.3)	
N2	10(25)	12(40)	
N3	8(20)	4(13.3)	
**Metastasis**			
Yes	11(27.5)	9(30)	0.819
No	29(72.5)	21(70)	
**AJCC stage**			
1	4(10)	5(16.7)	0.791
2	8(20)	4(13.3)	
3	17(42.5)	12(40)	
4	11(27.5)	9(30)	

Abbreviations: NSCLC: non-small cell lung cancer; NSCLC-D: NSCLC-at first diagnosis; NSCLC-R: NSCLC-at first relapse; ECOG PS: Eastern Cooperative Oncology Group performance status; SCC: squamous cell carcinoma; ADC: adenocarcinoma; AJCC: American Joint Committee on Cancer.

### The proportions of lymphocyte subsets in NSCLC patients

**Figure 1:**
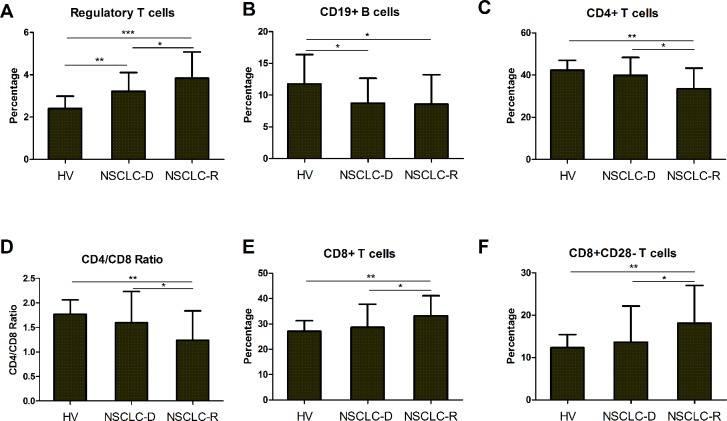
The proportions of lymphocyte subsets in NSCLC patients **A**. Increased regulatory T cells in NSCLC-D and NSCLC-R; Decreased **B**. CD19+ B cells, **C**. CD4+ T cells, and **D**. CD4/CD8 ratio; Elevated **E**. CD8+ T cells and **F**. CD8+CD28- T cells. Abbreviations: NSCLC = non-small cell lung cancer; HV = healthy volunteers; NSCLC-D = NSCLC-at first diagnosis; NSCLC-R = NSCLC-at first relapse. *P < 0.05; ** *P* < 0.01; *** *P* < 0.001.

**Figure 2:**
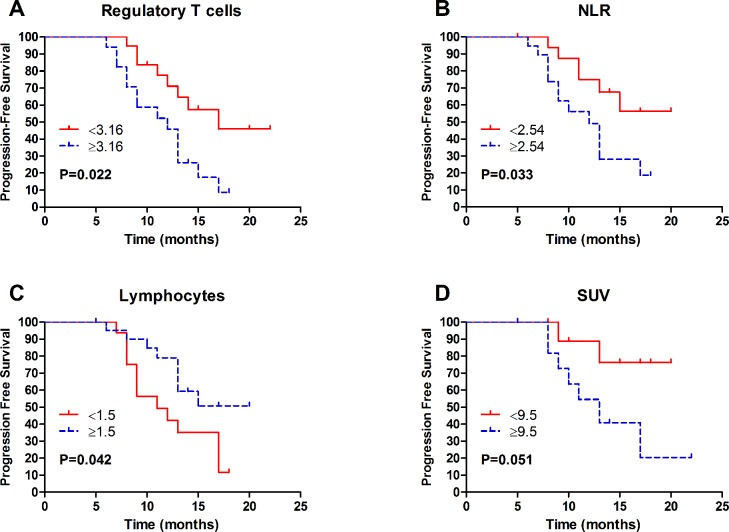
Kaplan-Meier of progression-free survival of 40 first diagnosed NSCLC patients according to A Regulatory T cells; **B**. NLR; **C**. Lymphocyte count; **D**. SUV. Abbreviations: NSCLC = non-small cell lung cancer; NLR = neutrophil/lymphocyte ratio; SUV = standard uptake value.

The percentage of Treg cells in cancer patients were considerably elevated compared to healthy controls; the high expression of Treg cells was further validated in NSCLC patients at first relapse compared to newly diagnosed patients (Table 2, Figure 1A). In contrast, decreased proportions of CD19+ B cells, CD4+ T cells, and CD4/CD8 ratios were observed (Table 2, Figure 1B-1D). Besides, increased CD8+ T cells and CD8+CD28- T cells were showed in NSCLC patients (Table 2, Figure 2E-2F). However, there were no differences in NK, NKT, γδT, CD3+ and CD8+CD28+ T cells between patients and controls (Table 2). In 30 patients at first relapse, increased Treg cells were observed in metastatic patients compared to recurrent patients; decreased proportions of CD4+ T cells, CD4/CD8 ratios were presented with a statistical trend (Table 3).

**Table 2:** Comparisons of lymphocyte subsets between NSCLC patients and healthy controls

Immune parameter	Healthy control	NSCLC-D	NSCLC-R	*P*
CD3+ T cells	72.87±5.40	69.63±12.83	67.89±11.02	0.398
CD4+ T cells	42.26±4.70	39.85±8.49	33.53±9.74	**0.002**
CD8+ T cells	27.05±4.21	28.65±9.12	33.20±7.00	**0.025**
CD4/CD8 ratio	1.77±0.29	1.60±0.63	1.24±0.60	**0.008**
CD8+CD28+ T cells	14.59±2.70	13.72±4.92	13.44±3.86	0.708
CD8+CD28- T cells	12.32±3.10	13.64±8.52	18.11±8.92	**0.033**
Treg	2.40±0.59	3.22±0.89	3.83±1.24	**0.001**
CD19+ B cells	11.77±4.62	8.74±3.91	8.57±4.66	0.058
NK	16.24±6.33	20.22±11.92	20.98±8.91	0.350
NKT	6.85±4.20	5.54±4.63	6.37±4.22	0.590
γδT	5.62±4.66	5.17±7.08	5.11±4.42	0.963

Data were expressed as Mean±SD. Abbreviations: NSCLC: non-small cell lung cancer; NSCLC-D: NSCLC-at first diagnosis; NSCLC-R: NSCLC-at first relapse; SD: standard deviation; Treg: regulatory T cells.

**Table 3:** Differences of lymphocyte subsets between local recurrence and metastasis in 30 NSCLC patients at first relapse

Immune parameter	Local recurrence (*N*= 18)	Metastasis (*N*= 12)	*P*
CD3+ T cells	68.72±11.03	66.63±11.36	0.619
CD4+ T cells	37.42±11.18	32.14±9.08	0.184
CD8+ T cells	30.20±8.51	32.71±7.56	0.416
CD4/CD8 ratio	1.38±0.69	1.03±0.37	0.083
CD8+CD28+ T cells	13.09±4.10	13.97±3.56	0.548
CD8+CD28- T cells	17.52±9.83	19.00±7.70	0.665
Treg	2.61±0.98	3.55±1.21	**0.046**
CD19+ B cells	8.02±3.66	9.26±5.82	0.547
NK	19.66±8.90	22.65±9.06	0.418
NKT	6.36±5.34	6.38±2.39	0.990
γδT	4.97±4.45	5.29±4.60	0.864

Data were expressed as Mean±SD. Abbreviations: NSCLC: non-small cell lung cancer; SD: standard deviation; Treg: regulatory T cells.

### Correlation of lymphocyte subpopulations with clinicopathologic parameters

Table 4–5 summarizes the associations between lymphocyte subsets and clinicopathological variables in 40 newly diagnosed patients. Patients who were younger and with negative tumor markers had higher proportions of CD3+ T cells compared to their counterparts. Similarly, increased CD4+ T cells were observed in young patients. In females, there were elevated proportions of CD4+ T cells and CD4/CD8 ratios compared to males. High levels of CD8+CD28+ T cells were occurred in patients with negative tumor markers (Table 4). Nonsmokers showed increased γδT cells compared to smokers (Table 5). We found no significant associations between CD8+CD28- T cells, Treg, CD19+ B cells, NK, NKT cells and clinicopathologic characteristics.

**Table 4:** Correlation between T lymphocyte subsets and clinicopathologic characteristics in 40 newly diagnosed NSCLC patients

Parameter	CD3+ T cells	*P*	CD4+ T cells	*P*	CD8+ T cells	*P*	CD4/CD8 ratio	*P*	CD8+CD28+ T cells	*P*	CD8+CD28- T cells	*P*	Treg	*P*
**Sex**														
Female	72.86±12.42	0.303	44.04±8.04	**0.046**	26.64±12.39	0.749	2.11±1.30	**0.036**	12.41±3.42	0.277	13.55±9.26	0.965	3.42±1.20	0.562
Male	68.24±12.97		37.28±10.01		27.73±8.60		1.47±0.58		14.28±5.40		13.68±8.37		3.20±0.93	
**Age(years)**														
<60	75.11±10.36	**0.046**	44.03±7.74	**0.024**	28.65±10.00	0.559	1.72±0.67	0.769	14.19±3.95	0.662	13.43±8.19	0.909	3.45±1.18	0.414
≥60	66.68±13.24		36.76±10.08		26.73±9.73		1.63±1.00		13.47±5.43		13.76±8.86		3.16±0.89	
**Smoking**														
Never	71.78±12.19	0.445	40.15±10.07	0.697	26.37±11.80	0.628	1.92±1.24	0.180	10.71±3.15	**0.003**	15.51±10.83	0.315	3.06±1.05	0.426
Previous/present	68.47±13.25		38.85±9.94		27.96±8.64		1.53±0.62		15.34±4.99		12.63±7.03		3.35±0.98	
**ECOG PS**														
0	72.34±10.66	0.281	40.34±7.51	0.597	29.51±9.59	0.269	1.65±1.13	0.928	14.87±5.04	0.233	15.32±7.83	0.314	3.29±0.63	0.919
1-2	67.82±14.02		38.62±11.28		26.00±9.78		1.68±0.72		12.96±4.80		12.52±8.94		3.25±1.17	
**SUV**														
<9.5	69.51±14.92	0.949	39.21±9.44	0.479	27.68±10.70	0.982	1.76±1.36	0.582	12.96±4.21	0.364	14.85±8.32	0.557	3.00±0.90	0.600
≥9.5	69.11±14.22		36.00±11.09		27.58±10.73		1.51±0.68		11.30±4.18		17.38±10.98		2.81±0.66	
**Histology**														
SCC	66.76±15.13	0.228	36.29±10.57	0.138	27.33±9.87	0.779	1.48±0.60	0.366	13.48±4.90	0.711	14.21±9.81	0.866	3.05±0.97	0.422
ADC	71.92±10.37		40.88±7.89		28.24±10.07		1.74±1.09		14.10±5.30		13.72±7.53		3.31±0.91	
**Differentiation**														
Well/moderate	69.9±12.63	0.675	39.34±8.67	0.170	27.94±10.41	0.828	1.68±0.98	0.256	12.78±3.96	0.234	14.64±9.12	0.903	2.95±0.84	0.397
Poor	67.6±17.16		34.07±11.89		28.80±9.12		1.27±0.59		14.98±6.21		15.08±8.76		3.25±0.91	
**Tumor stage**														
T1	65.48±12.86	0.243	37.48±8.89	0.507	25.27±9.61	0.430	1.83±1.34	0.519	12.05±5.71	0.221	13.94±6.40	0.899	3.10±0.70	0.601
T2-T4	71.01±12.74		39.92±10.25		28.12±9.84		1.61±0.71		14.28±4.61		13.54±9.22		3.31±1.07	
**Nodal stage**														
N0	69.61±10.82	0.995	39.02±9.23	0.913	27.93±10.76	0.836	1.77±1.34	0.654	14.71±4.84	0.443	12.60±6.55	0.641	3.24±1.01	0.936
N1-N3	69.64±13.70		39.41±10.26		27.20±9.52		1.63±0.68		13.35±4.99		14.03±9.24		3.27±1.01	
**AJCC stage**														
1-3	68.79±12.97	0.508	38.83±10.07	0.626	26.90±9.39	0.598	1.67±0.94	0.950	13.43±4.49	0.555	13.61±8.35	0.974	3.21±0.98	0.614
4	71.85±12.79		40.56±9.69		28.75±10.96		1.65±0.80		14.48±6.11		13.71±9.38		3.39±1.07	
**Tumor Marker**														
Negative	75.53±8.82	**0.049**	41.98±6.86	0.241	32.44±9.88	**0.026**	1.46±0.68	0.294	15.91±5.14	**0.050**	15.58±8.00	0.288	3.27±1.18	0.960
Positive	66.41±13.64		38.43±10.82		24.62±9.13		1.81±0.98		12.45±4.56		12.27±8.75		3.25±0.97	

Data were expressed as Mean±SD. Abbreviations: NSCLC: non-small cell lung cancer; SD: standard deviation; Treg: regulatory T cells; SUV: standard uptake value; ECOG PS: Eastern Cooperative Oncology Group performance status; SCC: squamous cell carcinoma; ADC: adenocarcinoma; AJCC: American Joint Committee on Cancer.

**Table 5:** Correlation between other lymphocyte subsets and clinicopathologic characteristics in 40 newly diagnosed NSCLC patients

Parameter	CD19+ B cells	*P*	NK	*P*	NKT	*P*	γδT	*P*
**Sex**								
Female	11.72±6.13	0.057	14.49±10.65	0.073	4.53±2.81	0.423	4.13±2.61	0.590
Male	8.36±3.90		22.43±11.83		5.93±5.16		5.58±8.19	
**Age(years)**								
<60	9.54±5.90	0.823	15.74±10.05	0.090	5.71±2.92	0.873	5.14±3.56	0.986
≥60	9.16±4.15		22.75±12.35		5.45±5.43		5.19±8.53	
**Smoking**								
Never	9.60±6.39	0.802	17.54±10.62	0.377	6.70±7.12	0.330	9.60±11.59	**0.011**
Previous/present	9.16±4.03		21.41±12.47		5.04±3.06		3.23±2.04	
**ECOG PS**								
0	8.22±2.63	0.318	18.31±10.13	0.476	4.24±2.61	0.211	4.09±2.26	0.498
1-2	9.90±5.61		21.31±12.91		6.28±5.38		5.79±8.71	
**SUV**								
<9.5	7.84±3.59	0.728	22.00±15.39	0.854	4.11±1.70	0.145	4.71±2.61	0.427
≥9.5	7.25±3.60		20.89±10.41		8.08±7.60		8.26±12.82	
**Histology**								
SCC	8.45±3.77	0.435	23.58±12.86	0.138	5.58±6.09	0.933	5.43±9.49	0.865
ADC	9.52±4.14		17.41±10.46		5.72±2.72		5.00±3.82	
**Differentiation**								
Well/moderate	8.83±3.40	0.579	19.86±10.23	0.455	5.26±5.28	0.364	5.68±8.67	0.892
Poor	7.92±5.28		23.80±17.87		7.15±3.81		5.24±4.15	
**Tumor stage**								
T1	9.32±3.80	0.989	24.93±11.68	0.210	4.09±1.84	0.323	3.88±3.48	0.564
T2-T4	9.29±5.09		18.88±11.85		5.96±5.11		5.54±7.82	
**Nodal stage**								
N0	9.04±3.45	0.860	18.96±10.72	0.719	5.11±2.52	0.751	3.67±2.63	0.470
N1-N3	9.38±5.20		20.65±12.46		5.69±5.18		5.67±8.02	
**AJCC stage**								
1-3	9.02±4.85	0.607	21.70±12.37	0.269	4.87±4.96	0.193	5.61±8.11	0.582
4	9.92±4.78		16.87±10.60		7.07±3.51		4.17±4.02	
**Tumor Marker**								
Negative	10.26±3.68	0.534	14.59±8.79	0.054	5.89±2.69	0.785	3.53±2.32	0.434
Positive	9.09±5.34		23.24±12.40		5.39±5.41		5.71±8.50	

Data were expressed as Mean±SD. Abbreviations: NSCLC: non-small cell lung cancer; SD: standard deviation; Treg: regulatory T cells; SUV: standard uptake value; ECOG PS: Eastern Cooperative Oncology Group performance status; SCC: squamous cell carcinoma; ADC: adenocarcinoma; AJCC: American Joint Committee on Cancer.

### Increased Treg cells correlated with poor progression-free survival (PFS)

Table 6 summarizes the results of univariate analysis of lymphocyte subsets. High proportions of Treg cells correlated to worse PFS (HR = 2.55, 95%CI = 1.07-6.11, *P* = 0.022, Figure 2A). We found no significant association between other lymphocyte subsets with PFS. In addition to lymphocyte subpopulations, some other parameters were correlated with clinical outcomes (Table 6). The neutrophil/lymphocyte ratio (NLR) was negatively correlated to PFS (HR = 2.66, 95%CI = 1.01-7.05, *P* = 0.033, Figure 2B); conversely, a positive association was observed between lymphocytes and PFS (HR = 0.42, 95%CI = 0.17-1.03, *P* = 0.042, Figure 2C). Moreover, a high standard uptake value (SUV) correlated with poor survival, with a strong trend toward significance (HR = 4.14, 95%CI = 0.85-20.11, *P* = 0.051, Figure 2D). In terms of clinicopathologic characteristics, the Eastern Cooperative Oncology Group performance status (ECOG PS), tumor differentiation, clinical stage, and nodal stage correlated with clinical outcome of NSCLC patients (Table 6).

**Table 6:** Univariate analysis of progression-free survival of 40 newly diagnosed NSCLC patients according to clinicopathologic characteristics and lymphocyte subsets

Parameter	Median survival (months)	HR (95%CI)	*P*
**Age (≥60** *vs* **<60)**	17 *vs* 13	0.78(0.33-1.84)	0.551
**Sex (Male** *vs* **Female)**	13 *vs* NA	1.94(0.70-5.37)	0.173
**ECOG PS (1-2** *vs* **0)**	11 *vs* 16	3.59(1.30-9.94)	**0.006**
**Smoking (Previous/present** *vs* **Never)**	13 *vs* 13	1.33(0.54-3.27)	0.521
**SUV (≥9.5** *vs* **<9.5)**	13 *vs* NA	4.14(0.85-20.11)	0.051
**Histology (ADC** *vs* **SCC)**	13 *vs* 15	1.24(0.51-3.01)	0.614
**Differentiation (Poor** *vs* **Well/moderate)**	8 *vs* 17	4.91(1.70-14.15)	**0.001**
**Tumor Marker (Positive** *vs* **Negative)**	13 *vs* 15	1.93(0.64-5.80)	0.213
**Tumor stage (T2-4** *vs* **T1)**	13 *vs* NA	1.70(0.57-5.03)	0.312
**Nodal stage (N1-3** *vs* **N0)**	12 *vs* 16	3.70(1.09-12.58)	**0.019**
**AJCC stage (4** *vs* **1-3)**	10 *vs* 17	3.21(1.37-7.50)	**0.003**
**Leukocyte (≥median** *vs* **<median)**	13 *vs* 13	0.96(0.38-2.38)	0.919
**Lymphocyte (≥1.5** *vs* **<1.5)**	14 *vs* 11	0.42(0.17-1.03)	**0.042**
**NLR (≥2.54** *vs* **<2.54 )**	12 *vs* 16	2.66(1.01-7.05)	**0.033**
**PLR (≥median** *vs* **<median)**	13 *vs* 13	1.34(0.55-3.29)	0.500
**MLR (≥median** *vs* **<median)**	15 *vs* 13	1.21(0.50-2.94)	0.655
**Treg (≥3.16** *vs* **<3.16 )**	12 *vs* 17	2.55(1.07-6.11)	**0.022**
**CD3+ T cells (≥median** *vs* **<median)**	13 *vs* 13	0.99(0.43-2.31)	0.989
**CD4+ T cells (≥median** *vs* **<median)**	13 *vs* 13	1.16(0.50-2.68)	0.722
**CD8+ T cells (≥median** *vs* **<median)**	17 *vs* 13	0.86(0.37-2.01)	0.720
**CD4/CD8 ratio(≥median** *vs* **<median)**	13 *vs* 17	1.53(0.65-3.60)	0.302
**CD19+ B cells (≥median** *vs* **<median)**	11 *vs* 13	1.13(0.49-2.61)	0.767
**NK (≥median** *vs* **<median)**	13 *vs* 13	0.97(0.42-2.27)	0.945
**NKT (≥median** *vs* **<median)**	11 *vs* 15	1.57(0.67-3.68)	0.271
**γδT (≥median** *vs* **<median)**	13 *vs* 15	1.49(0.63-3.49)	0.335
**CD8+CD28+ T cells (≥median** *vs* **<median)**	13 *vs* 13	0.82(0.35-1.90)	0.630
**CD8+CD28- T cells (≥median** *vs* **<median)**	17 *vs* 13	0.67(0.29-1.55)	0.325

Abbreviations: NSCLC: non-small cell lung cancer; HR: hazard ratio; CI: confidence interval; Treg: regulatory T cells; SUV: standard uptake value; SCC: squamous cell carcinoma; ADC: adenocarcinoma; ECOG PS: Eastern Cooperative Oncology Group performance status; AJCC: American Joint Committee on Cancer; NLR: neutrophil/lymphocyte ratio; PLR: platelet/lymphocyte ratio; MLR: monocyte/lymphocyte ratio.

### Elevated Treg cells independently predict poor PFS

On multivariate analysis, elevated Treg cells were independently correlated with poor PFS (HR = 3.76, 95%CI = 1.38-10.22, *P* = 0.010, Table 7). For well-recognized prognostic factors, the independent roles of American Joint Committee on Cancer (AJCC) stage and nodal stage were found as well.

**Table 7:** Multivariate analysis of progression-free survival of 40 newly diagnosed NSCLC patients

Parameter	HR (95%CI)	*P*
**Treg**		
<3.6	1	0.010
≥3.6	3.76(1.38-10.22)	
**Differentiation**		
Well/moderate	1	0.152
Poor	2.46(0.72-8.40)	
**AJCC stage**		
1-3	1	0.005
4	5.74(1.71-19.19)	
**Nodal stage**		
N0	1	0.004
N1-N3	8.12(1.94-33.98)	
**ECOG PS**		
0	1	0.101
1-2	2.10(0.65-6.75)	

Abbreviations: NSCLC: non-small cell lung cancer; HR: hazard ratio; ECOG PS: Eastern Cooperative Oncology Group performance status; Treg: regulatory T cells; AJCC: American Joint Committee on Cancer.

## DISCUSSION

We demonstrated that elevated Treg cells were independently associated with poor PFS in NSCLC patients received radiotherapy. In addition, NLR and the lymphocyte count were associated with tumor progression in univariate analysis. Moreover, we further validated the increased proportions of Treg cells, CD8+ T cells, and CD8+CD28- T cells and decreased CD4+ T cells and CD4/CD8 ratios in NSCLC patients at first relapse compared to newly diagnosed patients. To our knowledge, this is the first study to validate the predictive significance of Treg cells in patients with NSCLC undergoing radiotherapy, and the expression of them in patients at first relapse.

Treg cells suppress anti-tumor activity elicited by adaptive immune system in human cancer and facilitate tumor growth [21]. Several studies have proved the prognostic and predictive significance of peripheral Treg cells in lung cancer patients undergoing surgery and chemotherapy [15, 19]. In the present study, we further confirmed its predictive role in NSCLC patients treated with radiotherapy. Elevated proportions of Treg cells indicated rapid tumor progression after radiotherapy, which may because of the suppressor function of Treg cells and their resistance to radiation [22, 23]. In addition to lymphocyte subsets, we also observed that lymphocyte counts and NLR were correlated with survival without relapse, which were consistent with published studies [24, 25]. The predictive value of NLR, platelet/lymphocyte ratio (PLR) and monocyte/lymphocyte ratio (MLR) were previously investigated in various kinds of cancer [24, 26–28]. A recent study observed that pretreatment SUV correlated with PFS in early-stage NSCLC patients treated with SBRT [29]. We found a similar trend in the 22 patients whose SUV were available, although 7 of them had been treated with conventional fraction radiotherapy.

As immunosuppressive cells, increased Treg cells were observed in various tumors and metastatic diseases, including those with NSCLC [11, 30–32]. Our findings are consistent with these observations and supported this role in patients at first relapse compared to newly diagnosed patients. In addition, we found that NSCLC patients with metastatic disease showed high level of Treg cells compared to local recurrence, which may suggest the relationship between increased Treg cells and tumor progression. However, we found no differences of other lymphocyte subsets between metastatic disease and local recurrence, which may be attributable to the limited number of patients. Our study showed decreased proportions of CD4+ T cells, CD4/CD8 ratio and CD19+ B cells in NSCLC patients, which is in agreement with previous studies [12, 33]. As important immune cells that prevent tumor progression, CD8+ cytotoxic T lymphocytes (CD8+CD28+ T cells) were decreased in NSCLC patients compared to healthy volunteers although it did not reach significance. CD8+CD28- T cells, exhibiting immunosuppressive role, were significantly increased in NSCLC patients, which may contribute to the increase of CD8+ T cells. Furthermore, the expression of these immune cells was confirmed in NSCLC patients at first relapse compared to patients at first diagnosis. These observations may imply the important role of adaptive immune system in cancer patients.

Younger patients, females and those with negative tumor markers presented increase of CD4+, CD8+CD28+ T cells, and CD4/CD8 ratios in the periphery, which is consistent with a previous study [34]. However, we have not observed significant correlation between these lymphocyte subsets with tumor stage, which may attribute to the limited patients number.

There are several limitations in our study. First, the sample size is small. Second, the clinical stages of patients in our study are not homogeneous. Third, different radiation doses and fractions were used. Despite these limitations, we proved that the level of Treg cells independently predicted tumor progression. Moreover, increased Treg cells were observed in NSCLC patients at first diagnosis and at first relapse compared to healthy controls.

## CONCLUSIONS

In NSCLC patients undergoing radiotherapy, the level of peripheral Treg cells may serve as a useful predictor of tumor progression, indicating that adaptive immunity is highly important for NSCLC patients. Those findings suggest that radiotherapy combined with the manipulation of Treg cells may become a successful treatment for NSCLC patients.

## MATERIALS AND METHODS

### Patients and clinical data

Seventy histologically confirmed NSCLC patients were prospectively enrolled in the present study, consisting of 40 newly diagnosed patients and 30 patients at first relapse. The 40 newly diagnosed patients were over 18 years old and had not received treatment for cancer before enrollment. They had no immune system related diseases, no infections, transplant history, cancer of other types, and they had not received steroid treatment before enrollment. The 30 NSCLC patients at first relapse had not received anti-tumor therapy, immunotherapy and steroid therapy for at least 3 months (exclude one who received gefitinib) before enrollment. Fourteen age-matched healthy controls were enrolled. All volunteers and patients provided informed consent. This study was approved by the Ethical Committee of The Affiliated Hospital of Academy of Military Medical Sciences.

We obtained patient characteristics from electronic records. Tumor stage was evaluated by [AJCC]-7 criteria [35]. Tumor markers for NSCLC contained squamous cell carcinoma antigen (SCC), carcinoembryonic antigen (CEA) and cytokeratinfragment (CYFRA 21-1). Tumor marker was positive when either of them was positive. NLR, PLR and MLR were collected from peripheral blood routine test.

### Blood samples and flow cytometry

Fresh blood samples were collected from patients prior to anti-cancer treatment and from volunteers. Ten specific monoclonal antibodies (mAbs) against CD3 (APC and PerCP), CD4 (FITC and APC), CD8 (FITC and APC), CD16 (PE), CD19 (APC), CD25 (APC), CD28 (PE), CD56 (PE), CD127 (PE), and TCR (PE) were used to differentiate lymphocyte subsets. At the beginning, we mixed 100 μl fresh blood with the above mAbs and incubated at room temperature for 15 minutes in the dark. We used FACS lysing solution (BD Biosciences, San Jose, CA, USA) to lyse red blood cells in the mix and then washed twice with phosphate buffered saline (PBS). After that, flow cytometry was used to analysis the residual white blood cells and the proportions of the lymphocyte subsets were calculated by FlowJo Version 10 data analysis software (FlowJo, Ashland, OR, USA).

Lymphocyte subpopulations were identified as follow: CD3+ T cells (CD3+CD19-), CD4+ T cells (CD3+ CD4+CD8-), CD8+ T cells (CD3+CD8+CD4-), CD8+CD28+ T cells (CD3+CD8+CD28+), CD8+CD28- T cells (CD3+CD8+CD28-), Treg (CD4+CD25+CD127low), B cells (CD3-CD19+), natural killer (NK) cells (CD3-CD16+CD56+), natural killer T (NKT) cells (CD3+CD16+CD56+), gamma delta T (γδT) cells (CD3+TCR+). Lymphocyte subsets were determined by the percentages of total lymphocytes.

### Treatment and follow up

The 40 newly diagnosed NSCLC patients received radiotherapy alone or combined with targeted therapy/chemotherapy concurrently or consecutively. Radiotherapy contained SBRT with 30-50 Gy/5 fractions by Cyber Knife (Accuray, Sunnyvale, CA, USA) and conventional fraction radiotherapy with 60-66 Gy/30-33 fractions using linear accelerator. Platinum-based agents were used for first-line chemotherapy; crizotinib, erlotinib, and gefitinib were used for targeted therapy.

Follow-up was ended on October 20, 2016 or the time of death of the patient. Patients were followed up regularly every 3 months after radiotherapy. The median follow-up was 12.5 months (range: 5-22 months). PFS was the primary endpoint, which calculated from the time of initial treatment to the time of first progression defined by RECIST (Response Evaluation Criteria in Solid Tumors) 1.1 [36] criteria, loss to follow-up, or death.

### Statistical analysis

Proportions of lymphocyte subpopulations were expressed with mean±standard deviation (SD). Basic characteristics between patients at first diagnosis and relapse were compared by Chi-square and Fisher exact tests. Differences of immune parameters between the two groups of lung cancer patients and the controls were evaluated by one-way analysis of variance (ANOVA) and post-hoc multiple comparisons. The Student’s *t-*test was used to determine relationships between lymphocyte subsets and patient characteristics. We employed the Kaplan-Meier analysis to estimate PFS and log-rank test to compare the survival of two groups. The Cox proportional hazards model was used to determine hazard ratios (HRs) and 95% confidence intervals (CIs). Considering the limited number of patients, only parameters of *P* < 0.025 in univariate analysis were included in multivariate analysis. We used the Statistical Package for Social Sciences, Version 20.0 (IBM Corporation, Armonk, NY, USA) to analyze the data. *P* value < 0.05 was considered statistically significant.
